# Calories Do Not Explain Extension of Life Span by Dietary Restriction in *Drosophila*

**DOI:** 10.1371/journal.pbio.0030223

**Published:** 2005-05-31

**Authors:** William Mair, Matthew D. W Piper, Linda Partridge

**Affiliations:** **1**Centre for Research on Ageing, University College London, Department of Biology, LondonUnited Kingdom; University of Newcastle upon TyneUnited Kingdom

## Abstract

Dietary restriction (DR) extends life span in diverse organisms, including mammals, and common mechanisms may be at work. DR is often known as calorie restriction, because it has been suggested that reduction of calories, rather than of particular nutrients in the diet, mediates extension of life span in rodents. We here demonstrate that extension of life span by DR in *Drosophila* is not attributable to the reduction in calorie intake. Reduction of either dietary yeast or sugar can reduce mortality and extend life span, but by an amount that is unrelated to the calorie content of the food, and with yeast having a much greater effect per calorie than does sugar. Calorie intake is therefore not the key factor in the reduction of mortality rate by DR in this species.

## Abstract 2

Experimental evidence reveals that specific nutritional components, rather than reducing calorie intake per se, are responsible for extending lifespan via dietary restriction in *Drosophila melanogaster.*

## Introduction

Dietary restriction (DR), the extension of life span by reduction of nutrient intake without malnutrition, is often used as a benchmark comparison for interventions that extend life span [1–3]. Since McCay's pioneering experiments in rats 70 years ago [4], some form of food restriction has been shown to increase life span in commonly used model organisms such as yeast [5,6], nematodes [7], fruit flies [8,9], and mice [10], along with many species less often used for laboratory research such as water fleas, spiders, fish (see [3] for review), and dogs [11]. Preliminary data also suggest that DR may extend life span in nonhuman primates [12,13] and potentially give health benefits in humans [14]. Despite the finding that restricting diet increases longevity in such a diversity of species, the mechanisms responsible remain to be fully elucidated in any of them. It is therefore as yet unclear whether these mechanisms are evolutionarily conserved across taxa or if instead life extension during DR is an example of convergent evolution.

DR is often termed ‘calorie restriction' because, in rodents, daily calorie intake per se has been implicated as the key determinant of life span, with the source of these calories (i.e., carbohydrate, protein, or fat) being considered irrelevant [1]. Evidence for this point of view came from two types of experiment on rats: (1) restriction of calorie intake without reduction of protein intake resulted in life-span extension [15]; (2) no life-span extension was seen in rats fed isocaloric diets in which either the fat or mineral components had been reduced [16]. However, in other experiments, rats fed isocaloric diets with altered nutritional composition [17,18] or reduced protein [19] showed life-span extension. Furthermore, reducing just one amino acid (methionine) increases life span in both mice (R. Miller, personal communication) and rats [20]. Hence, it seems that reducing the level of ingested calories may not always be critical for life-span extension by DR in rodents. Here we address this issue in the fruit fly *Drosophila melanogaster.*

## Results

DR can be applied in *Drosophila* by the simultaneous dilution of the nutrients in a standard sugar yeast (SY) food medium [9] in which the yeast is the only source of protein and lipid. As food concentration declines from maximum, life span first increases in response to DR, becoming greatest at an intermediate food concentration, before declining due to starvation at lower concentrations [9,21]. We tested the separate effects of sugar and yeast on life span at the concentrations that maximise life span (DR) and under full feeding (control).

### Feeding Rates of Flies on Different Food Types

**Figure 1:**
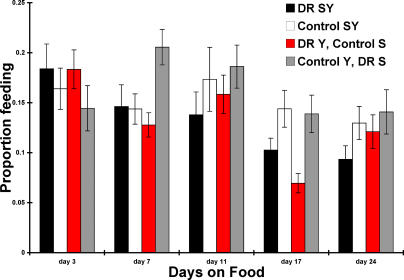
Feeding Rates of Female *Drosophila* on Food Media with Different Nutrient Concentrations Feeding rates were recorded by direct observation as the proportion of time flies spent on the surface of the media with their proboscis extended and touching the food (*y*-axis). Replicate measurements of the proportion of females feeding versus those not feeding were recorded during a 2-h period on the days shown. No significant different was seen between flies fed different diets on days 3, 7, 11, and 24 as assessed by chi-squared tests (*p* > 0.01, Bonferroni correction for multiple comparisons). There was a significant difference in feeding rates on day 17 (*p* = 0.0068) with flies on the DR yeast/control sugar media eating less. These data show that *Drosophila* does not exhibit compensatory feeding behaviour for the DR regime imposed.

Because flies may respond to changes in dietary composition by altering their feeding behaviour, thereby potentially compensating, we determined the effect of food composition on the amount of time that the flies spent feeding on different diets. Varying the proportions of sugar or dead yeast fed to adult *Drosophila* females did not have a significant effect on feeding behaviours (Figure 1; *p* > 0.01 in all cases, chi-squared test, Bonferroni adjustment for multiple comparisons). A significant difference was seen on day 17 (chi-squared, *p* = 0.0068) with flies on DR yeast/control sugar food eating less. However, this difference was in the opposite direction to that expected if flies on low-nutrient diets compensated by increasing feeding rates. Hence, the flies did not compensate for decreased nutrient content of the food medium by increasing the time that they spent feeding.

### Caloric Content of Dead Yeast/Sucrose

Values for yeast biomass components were taken from Lange and Heijnen [22] and estimations of the caloric content of protein, carbohydrate and lipid from Southgate and Durnin [23]. This allowed estimation of the caloric content per gram of sucrose and autolysed yeast powder, the only sources of nutrients in the *Drosophila* food medium. These values were 4 kcal/g sucrose and 4.02 kcal/g autolysed yeast powder. Since these values are virtually identical, changing either the sugar or yeast content of the foods between the DR and control concentrations generated food types with similar caloric values but with different nutritional compositions (see Table 1).

**Table 1:**
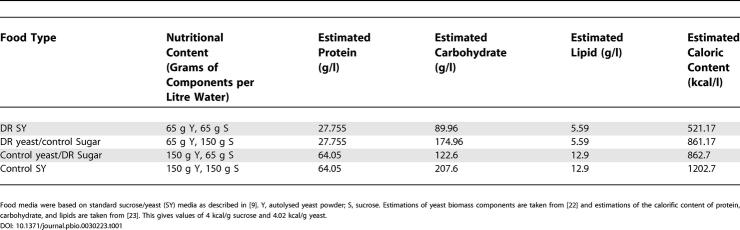
Nutritional Composition and Caloric Content of Experimental Food Types

### Life Span of Female *Drosophila* Given Foods of Different Caloric Value

**Figure 2:**
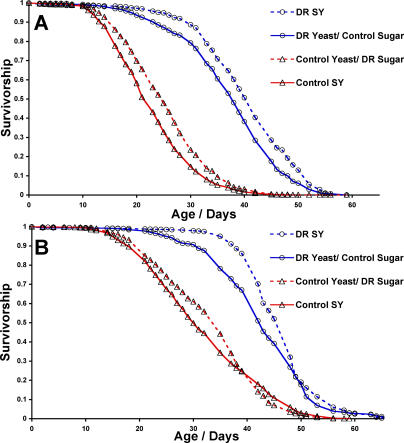
Survivorship (l*_x_*) Analysis of Life Span of Female *Drosophila* on Different Food Regimes Colour/Symbol of the curves shows yeast level while the line type represents sugar levels in the respective foods. (A) and (B) are independent repeats. In both cases, changing caloric content of the food by altering yeast levels had a much greater effect on life span than that seen when the same change in caloric content was brought about by manipulating sugar levels.

**Figure 3:**
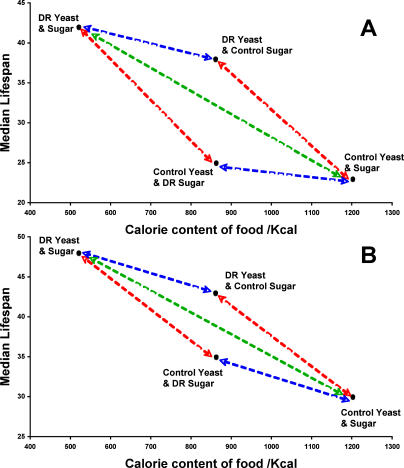
Plot of Median Life Span of Female *Drosophila* against the Estimated Caloric Content of the Food Medium (A) and (B) represent independent repeats. Red arrows link pairs of food types where differences in caloric content are due to different yeast concentrations. Blue arrows link pairs of food types where differences in caloric content are due to different sugar concentrations. Green arrow links food types where differences in caloric content are due to both different sugar and yeast concentrations. Life span is extended to a greater extent per calorie by reducing yeast concentration from control to DR levels than by reducing sugar. This is in contrast to what would be predicted if calorie intake were the key mediator of life-span extension by DR in fruit flies.

Life span of female *Drosophila* was extended much more by reduction of yeast from control to DR concentration than by the equivalent reduction in sugar (Figure 2; Table 2), and median life span therefore did not correlate with caloric content of the food medium to which the flies were exposed (Figure 3). In two independent experiments, reducing yeast concentration from control to DR levels whilst keeping sugar levels constant significantly increased life span (*p* < 0.0001 in both cases, log-rank test). Lowering caloric content to the same extent by reducing sugar from control to DR levels increased life span at DR yeast levels in both experiments (*p* < 0.0001 in both cases, log-rank test), but the effect on median life span was much less than that of changing yeast levels (Figure 3; Table 2). Reducing sugar from control to DR concentrations whilst keeping yeast at control levels significantly increased life span in experiment 1 (p < 0.0001, log-rank test), but again the effect on median life span was much less than that of changing yeast levels (Figure 3; Table 2). Reducing sugar from control to DR concentrations whilst maintaining yeast at control levels increased median life span in experiment 2 (Figure 3), but the effect on life span was not significant (*p* > 0.05, log-rank test).

**Table 2:**
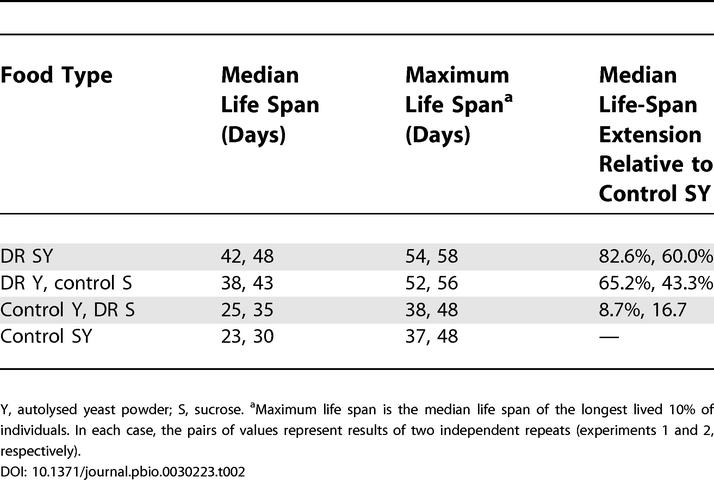
Median and Maximum Life Span of Flies Fed Different Food Media as Adults

### Effect of Bacteria on Response of Life Span to Diet

**Figure 4:**
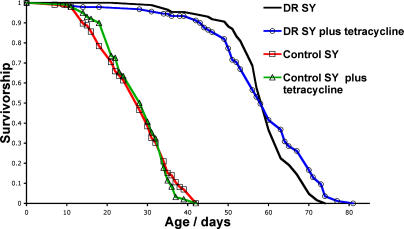
Effect of Tetracycline on Life Span of Female *D. melanogaster* The addition of the antibiotic tetracycline to the food media did not have a significant effect on life span at either control or DR concentration food media.

To test if different levels of bacteria in the food medium could account for effects of nutrient composition on life span, we tested the effect of an antibiotic. The addition of the antibiotic tetracycline to the food media did not have a significant effect on life span on either control or DR food medium (Figure 4, *p* > 0.05; log-rank test in each case), and the life-span extension seen when sugar and yeast levels were reduced from control to DR concentrations was therefore not blocked or modified by the addition of antibiotic to the food medium.

### Effects on Mortality of Switching Yeast and Sugar

**Figure 5:**
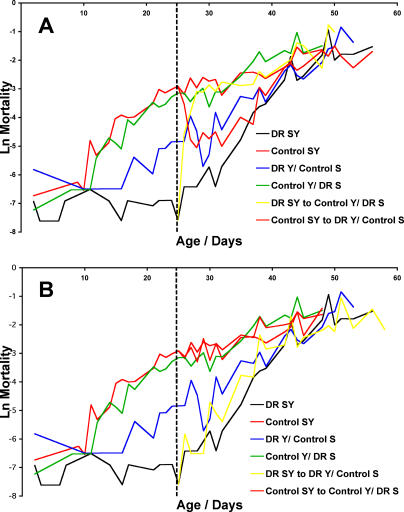
The Acute Effects on Age-Specific Mortality in *Drosophila* of Changes in Nutritional Content of the Food Midway through Life Vertical line represents switch day. Mortality trajectories were truncated when *n* < 40. (A) Switching between control and DR yeast (Y) diets midway though life results in rapid changes in age-specific mortality rates within 48 h similar to those seen previously for whole food dilutions [24]. Control yeast intake caused no irreversible damage since flies switched from control yeast to DR yeast at day 25 rapidly became no more likely to die than those flies given DR yeast levels throughout adulthood. Flies with a history of DR yeast levels showed rapid increases in mortality rate when moved to control yeast levels at day 25, but mortality rates did not become as high as those of flies that had been maintained on control yeast levels permanently. (B) Changing caloric intake to the same extent via changes to sugar (S) levels rather than yeast did not cause rapid changes in mortality rate. Despite flies chronically fed control sugar and DR yeast having increased mortality rate compared to the DR control, switching from DR to control sugar late in life did not increase mortality rate.

The effect of DR on mortality in *Drosophila* is acute; within 48 h flies switched between DR and control diets adopt the mortality rates characteristic of flies chronically exposed to the nutritional regime that the switched flies have joined [24]. We therefore measured the acute effects on mortality of switching the yeast and sugar components of the diet separately. When yeast was switched, mortality rates responded similarly to the responses to switches between control and DR SY food medium. Forty-eight hours after being switched from control SY medium to DR yeast/control sugar medium at day 25, flies were no more likely to die than those maintained on DR yeast/control sugar medium throughout adulthood (Cox regression; *p* = 0.22; *n* DR yeast/control sugar chronic group = 626; *n* switch group = 475; risk ratio = 0.96 [95% confidence interval CI: 0.91, 1.02]) (Figure 5A). In the reciprocal switch, flies moved from DR SY medium to control yeast/DR sugar medium showed a rapid increase in mortality rate, although this did not quite reach the level seen in flies that had been on control yeast/DR sugar medium throughout adult life (Cox regression; *p* < 0.05; *n* control yeast/DR sugar chronic group = 480; *n* switch group = 668; risk ratio = 0.88 [95% CI: 0.83, 0.93]) (Figure 5A).

In contrast, switching of sugar had no significant effect on mortality. From 48 h after being switched from control SY medium to control yeast/DR sugar at day 25, no significant difference was seen between the mortality of switched flies and the unswitched group maintained on control SY medium (Cox regression; *p* = 0.34; *n* control SY = 427; *n* switch group = 440; risk ratio = 0.97 [95% CI: 0.91, 1.04]) (Figure 5B). Similarly, flies switched to DR yeast/control sugar from DR SY medium at day 25 did not show increased mortality in comparison to unswitched controls (Cox regression; *p* = 0.41; *n* DR group = 615; *n* switch group = 676; risk ratio = 0.98 [95% CI: 0.93, 1.03]) (Figure 5B). A second experiment that was terminated 4 d after the switch in diet gave the same result (see Figure S1). These data show that the rapid switch in mortality rates upon changes between DR and control food medium are overwhelmingly attributable to the yeast rather than to the sugar component of the diet.

## Discussion

### Life Span Is Not Related to Calorie Intake

Flies fed food media with very similar caloric content showed marked differences in their life spans (see Figure 3). This finding is in direct contrast to what would be predicted if ingested calories were the key mediator of life span in *D. melanogaster* and demonstrates that the nutritional composition of the diet affects life-span extension by DR in this species. Reduction in the concentration of either sugar or yeast levels increased life span (see Figures 2 and 3). However, the magnitude of the effects on life span when the caloric content of the food was changed via altering yeast concentration far exceeded that seen when calories were changed to the same extent via manipulation of sugar levels, suggesting that protein/lipid levels have a greater effect on *Drosophila* survival than does carbohydrate. The differing effect of sugar and yeast on mortality in *Drosophila* could occur if different pathways sense nutrients during DR, possibly with different outputs affecting life span. Sir2 [25,26], Rpd3 [27], the insulin/IGF-like signalling [28], and target of rapamycin pathways [29] have all been implicated in mediating the response of life span to DR in *Drosophila,* with the latter two suggested to interact in the fly to control growth in response to nutrient levels [30]. The role of these and other candidate pathways in mediating the response of life span to specific nutrients should be investigated further. Sugar and yeast could affect mortality rates differently if they differentially modulate metabolic or other processes that increase risk of death.

Experimentally increased reproduction has been shown to decrease life span in a variety of species [31–35] and the level of dietary yeast and egg production are positively correlated in *Drosophila* [8,9]. Therefore an obvious hypothesis as to why there is a greater response of life span in *Drosophila* to changes in yeast than in sugar is that the increased mortality on control yeast levels represents the cost of reproduction, which correlates with yeast intake and not with sugar. However, since life-span extension via DR in *Drosophila* occurs normally when egg production or vitellogenesis are blocked either by X-irradiation or genetically [36], the greater response of life span to changes in yeast is not directly attributable to the reduction of reproductive output. Furthermore, although the magnitude of the response to DR in male *Drosophila* is less than that of females [21], males do live longer if nutrient levels are reduced, and they show the same rapid changes in mortality as females when dietary regime is changed [24], yet they do not suffer the high costs of producing eggs on high yeast.

### Rapid Changes in Mortality in Response to DR Are Attributable Solely to Yeast Content

DR acts acutely to extend life span in *Drosophila;* it does not slow the accumulation of irreversible damage with age [24]. Flies subjected to DR for the first time in midlife rapidly become no more likely to die than those that have been under DR throughout adulthood [24]. We investigated the roles of the sugar and yeast components of the diet in producing this rapid change in mortality rate in flies switched between DR and control conditions. When flies previously subjected to control SY food were switched to DR yeast levels, there was a rapid (within 48 h) drop in mortality rates to those seen in the flies chronically exposed to DR yeast/control sugar food (see Figure 5A). A similar rapid increase in mortality rates was seen when flies exposed to DR food were switched to control yeast levels (Figure 5A), although, as seen previously using whole food dilutions [24], a history of low yeast gave slight protection to female *Drosophila* moved to control yeast late in life.

However, when caloric content of the food given to flies was changed to the same extent midway though life by changing sugar rather than yeast levels, no change in mortality rate was seen (Figure 5B). Therefore the acute mortality ‘switch' phenotype in response to dietary restriction is attributable to changes in the level of the dietary yeast alone. That chronically reducing sugar intake of flies can extend life span, yet reducing sugar intake late in life does not cause rapid changes to mortality rates, suggests the deleterious effects of sugar may occur mainly early in adult life. The mortality trajectories in Figure 5 support this conclusion, by showing that the lowering of mortality rate in response to sugar is most obvious early in the trajectory, when mortality rates in all groups of flies are low. More work is needed using accurately defined media to investigate this effect. Rapid reductions in mortality rate have been seen previously in *Drosophila* by altering the intake of yeast only [37]. However, the results of the previous study differ from those here in that reduced mortality was achieved by *increasing* the nutrient intake of flies that had previously been deprived of yeast, rather than by reducing the nutrient intake of control-fed flies.

### Feeding Rates of Flies on Different Food Types

Unlike in rodents, where DR can be achieved by directly reducing the quantity of food eaten in comparison to animals given ad libitum access [1], DR is achieved in *Drosophila* by reducing the quality (nutrient concentration) of the food given to the flies [9] with the quantity maintained in excess of that which they can consume. Despite the fact that fecundity correlates with food medium concentration [9], it has been suggested that flies may be able to compensate when faced with reduced nutrients by increasing feeding rates, and therefore they may not be dietarily restricted [38]. However, our results suggest that flies on low-quality media do not compensate by eating more, as measured by time spent on the food with the proboscis extended. It is possible *Drosophila* can alter the rate of food uptake per unit time that the proboscis is extended, in which case our indirect measurements would not detect these changes. More direct approaches to quantify feeding rates require radio-labelling the food [39] or the addition of coloured food dye [40], with uptake rates assessed upon short-term exposure to labelled food. However, our own unpublished observations show that flies moved to fresh food medium display elevated feeding behaviour that is unrepresentative of the steady-state situation and that leads to a highly nonlinear relationship between time and uptake of the food label. We hence used the behavioural measure described here, which better represents the normal feeding of the flies. Our feeding assay results, in combination with the reduced fecundity seen as food nutrient concentration is reduced, suggest that diluting the food medium results in a co-ordinate reduction in the intake of nutrients in *Drosophila* and therefore is a robust protocol for DR in this species.

### Effect of Tetracycline on Life Span

It has been suggested that higher nutrient concentrations in fly food may lead to higher proliferation rates of bacteria on the media, which in turn could increase mortality of *D. melanogaster* in a mechanism that is unrelated to ingestion of different amounts of nutrients [38]. If this were the case then we would expect that (1) flies fed antibiotics would live longer, and (2) the life-span extension seen when nutrient concentration is reduced would be blocked when antibiotics are present. Tetracycline did not extend the life span of flies in our experiments, nor did it block the DR response, meaning either that reduced bacterial challenge is not the mechanism by which diluting food media extends life span in *Drosophila,* or that the relevant microorganisms are tetracycline resistant.

### Conclusions

The response of *Drosophila* life span to nutrition is not governed by calories, but rather by specific nutritional components of the food. This finding represents a departure from the generally accepted model in rodents, where it has been suggested that the level of calorie intake per se, not the source of calories, is critical for life-span extension [1]. The apparent disparity between the factors in the diet that affect life span in fruit flies and rodents leads to two possible conclusions. First, the mechanisms by which these organisms respond to food shortage could be different. Second, the long-held view that calorie intake is the critical variable in the response of mammalian life span to DR may require further evaluation.

Despite some reports in the literature that DR did not extend life span [38,41,42], the overwhelming majority of data support the idea that DR in some form extends life span across diverse taxa. However, it is still unknown if life-span extension under DR is achieved through common mechanisms in different species. A case for conservation of the mechanisms by which DR extends life span can be made from evolutionary considerations. It has been suggested that, during times of famine, diversion of resources away from reproduction towards somatic maintenance will increase the chances of an organism surviving to more plentiful times and thus increase long-term reproductive success [43–46]. The selective advantage of shifting resources from reproduction to maintenance when food is restricted could be the “public” factor shared between diverse organisms. However, the mechanisms by which extension of life span is achieved could be an example of convergent evolution, producing the same plasticity of life span in response to food shortage through mechanisms at least to some extent specific to different organisms, dependent upon their diet, experience of food shortages, and life history. More work is needed to elucidate the precise relationship between the composition of the diet and life span in different organisms, including mammals. Our results suggest that it may be possible to obtain the full extension of life span by DR by reducing critical nutrients in the food without any reduction in overall calorie intake.

## Materials and Methods

### Fly stocks and husbandry

The wild-type stock used in all experiments was collected in Dahomey (now Benin) in 1970 and has since been maintained in large population cages with overlapping generations on a 12:12-h light:dark cycle at 25 °C. This culturing method has been shown to maintain life span and fecundity at levels similar to those in freshly collected flies [47].

### Feeding rates of flies on different food types

To measure feeding rates in *Drosophila* we observed behaviour of age-matched, once-mated Dahomey females on each of the four food types. This approach was adopted in preference to direct quantification of ingested food [40] because DR flies transiently elevate their feeding rate following transfer onto new food (unpublished observations). In the present assay, 30 female flies were individually allocated to a vial containing either control SY, control Y/DR S, DR Y/control S, or DR SY and placed at 25 °C overnight to adopt their undisturbed pattern of feeding. The following day, 1 h after lights on, observations were taken for a 2-h period, and flies were scored as eating if they were on the food with their proboscis extended and touching the food surface. During this time, 360 observations of flies in each treatment were made (12 observations of 30 flies) except on day 24 when 18 observations were made of each treatment set. The final data are the proportion of flies feeding out of the feeding opportunities given (total observations). Differences between treatments at a given time point were assessed using the chi-squared test.

### Effect of tetracycline on life span

Tetracycline is a general antibiotic that inhibits ribosomal translocation and acts on both Gram-positive and negative bacteria [48]. A tetracycline solution was made up in 70% ethanol and added to the food media after it had been boiled and cooled to 60 °C. The final concentration of tetracycline in the media was 0.025% weight/volume [49], five times more than that used when tetracycline resistance is utilised as a selectable marker for bacterial transformation [50]. The wild-type stock Dahomey is infected by the cytoplasmic bacteria *Wolbachia* (unpublished). A 0.025% tetracycline solution is sufficient to remove bacteria such as *Wolbachia* from *Drosophila* stocks if fed to larvae [49] and can suppress *Wolbachia* in other insects when fed to adults only [51]. Therefore flies fed tetracycline media as adults may not only have reduced exposure to external microorganisms on the food surface compared to controls, but may also have reduced *Wolbachia* infection. Seven millilitres of food was poured into 30-ml glass vials and the life span of flies measured with 92–101 flies per treatment and 10 flies per vial. Fresh food was prepared once a week and flies moved onto new media three times per week.

### Life span experiments

Experimental flies were raised at a standard density of 400–450 eggs per 200-ml bottle [52] on standard SY medium (1,000 ml distilled water, 100 g autolysed yeast powder, 100 g sucrose, 20 g agar, 30 ml Nipagin (100 gl^–1^), 3 ml propionic acid). Adults were collected over a 24-h period and transferred without anaesthesia to fresh SY food for 48 h and allowed to mate. Females were then collected using light CO_2_ anaesthesia and assigned randomly to the food regimes (Table S1). All experiments were done with mated females. Flies were kept on 35 ml of food at an initial density of 100 individuals per 200-ml bottle and transferred without anaesthesia to fresh food every 2–3 d. Deaths were scored 5–6 d a week and initial sample sizes (*n*_0_) were calculated as the summed death and censor observations over all ages. To minimise any density effects on mortality, two bottles within cohorts were merged when the density of flies reached 50 ± 10. To standardise the effects of parental age on offspring fitness [53], parents of experimental flies were of the same age and reared at a constant density.

### Statistical analysis

Age-specific mortality (μ*_x_*) was estimated as μ*_x_* = −*ln(p_x_),* where *p_x_* is the probability of an individual alive at age *x* − 1 surviving to age *x* [54]. log-rank tests [55] were used for survivorship analysis. All statistical analysis was performed using JMP. 5.0 statistical software (SAS Institute Inc., Cary, North Carolina, United States).

## Supporting Information

Figure S1 The Acute Effects on Age-Specific Mortality in *Drosophila* of Changes in Nutritional Content of the Food Midway through Life Vertical line represents switch day. Experiment was terminated 4 d after the switch.
(A) Similar to the experiment shown in Figure 5, switching between control and DR yeast (Y) diets midway though life results in rapid changes in age-specific mortality rates within 48 h similar to those seen previously for whole food dilutions [24].
(B) Changing caloric intake to the same extent via changes to sugar (S) levels rather than yeast did not cause rapid changes in mortality rate.
(839 KB TIF).

Table S1 Sample Sizes and Treatments These represent the number of flies switched between treatments (i.e., *n*_25_) and were sampled from the original chronic controls (control SY or DR SY) and censored from the life-span data of these treatments at day 25.
(27 KB DOC).

## Abbreviations

CI: confidence interval
DR: dietary restriction
SY: sugar yeast
