# Supplementary material for: Calories Do Not Explain Extension of Life Span by Dietary Restriction in Drosophila
Source: PLoS Biol. 2005 May 31;3(7):e223. doi: 10.1371/journal.pbio.0030223 (PMC1140680; doi:10.1371/journal.pbio.0030223)
Supplement: Supplementary file 1 [file pbio.0030223.st001.doc]

| **Food Type (grams of components per litre water)** | **Experiment 1** | **Experiment 2** |
| --- | --- | --- |
| DR SY | 1000 | 2037 |
| DR Y, Control S | 962 | 674 |
| Control Y, DR S | 967 | 688 |
| Control SY | 957 | 2101 |
| Control SY until day 25 then Control Y, DR S * |  | 460 |
| Control SY until day 25 then DR Y, Control S * |  | 489 |
| DR SY until day 25 then Control Y, DR S * |  | 675 |
| DR SY until day 25 then DR Y, Control S * |  | 678 |
